# Effects of potassium fertilizer reduction combined with polyaspartic acid application on yield, quality, processing characteristics, rhizosphere microbial composition and metabolites of flue-cured tobacco

**DOI:** 10.3389/fpls.2025.1672845

**Published:** 2025-09-19

**Authors:** Haoran Zhang, Hui Tian, Zetao Zhang, Guohua Chai, Xiuwen Wu

**Affiliations:** ^1^ College of Resources and Environmental Sciences, Qingdao Agricultural University, Qingdao, China; ^2^ Laboratory Management Center, Qingdao Agricultural University, Qingdao, China

**Keywords:** PASP, K reduction, tobacco, chemical quality, physical properties, metabolites

## Abstract

**Introduction:**

The extensive application of potassium (K) in tobacco has exacerbated the shortage of K mineral resources in China. Polyaspartic acid (PASP) had shown significant effects in promoting the absorption of K, increasing the utilization rate of K fertilizer, and improving yield and quality of tobacco.

**Methods:**

To clarify the potential and mechanism of PASP replacing some K fertilizers in tobacco production, field experiment was conducted with 4 different treatments: NK (traditional fertilization); RT (10% reduction of K fertilizer); NKP (traditional fertilization +4% PASP/total K_2_O); RKP (10% reduction of K fertilizer +4% PASP/total K_2_O).

**Results:**

The reduction of K inhibited leaf growth, yield formation, K absorption, improved stem rate and decreased thickness, tensile strength, elongation rate and leaf surface density of flue-cured leaves, while had no effect on N, P, Cl content and rate of total sugar and nicotine. Comparing to NK, NKP raised the total yield by 16.7%, promoted physical properties and chemical quality optimization of flue-cured leaves. What is more, RKP compensated for the decrease in leaf yield, K content, processing utilization rate and sugar/nicotine of different parts caused by K reduction. However, K reduction and PASP application obviously influenced metabolite composition of rhizosphere soil without affecting the microbial community composition, and our results showed a relatively high correlation between differential metabolites and yield, K content, sugar/nicotine ratio, elongation rate of flue-cured tobacco.

**Conclusion:**

Overall, PASP provided better optimization effects on tobacco growth, yield, physical properties, chemical quality of flue-cured leaves, and PASP was beneficial for replacing K fertilizer to a certain extent, reducing the consumption of K mineral resources and environmental pollution.

## 1 Introduction

As one of the three essential elements of plant nutrition and fertilization, potassium (K) plays a crucial role in crop growth, development, especially in quality. However, excessive K application has become a major issue in Chinese crop production, which caused soil salinization, poor root growth, nutrient imbalance, and physiological diseases (Chen et al., 2005; Farhat et al., 2013; Zhu et al., 2015). China faces a shortage of potassium mineral resources, with over 50% of K fertilizer relying on imports to meet domestic demand. Thus, reducing K fertilizer use is essential for achieving sustainable agricultural development in China.

Tobacco (*Nicotiana tabacum* L.) is one of the main economic crops in China, with the largest planting area and yield in the world (Chen et al., 2022). As a K-demanding crop, tobacco requires substantial K fertilizer throughout its growth cycle (Farrokh et al., 2012). Potassium plays an important role in promoting tobacco growth, yield formation, and improving stress resistance, chemical quality, aroma, and combustibility (Marchand and Bourrie, 1997; Fu et al., 2022). It has been reported that the amount of K fertilizer used in tobacco was as high as 400 kg/hm^2^ in the southeastern China (Zhang et al., 2009), which has led to problems such as reduced nicotine content in tobacco leaves, decreased K fertilizer utilization rate, and soil compaction (Zhong et al., 2008; Li et al., 2024).

Polyaspartic acid (PASP), as a “green” fertilizer enhancer composed of amino acids, has been widely applied in agricultural production due to its advantages of strong biocompatibility, biodegradability, and environmental friendliness (Deng et al., 2014; Liu et al., 2022). Numerous studies have shown a significant positive correlation between PASP and K absorption of plants: exogenous application of PASP promoted the K content in tomato roots and stems (Hu et al., 2019); the accumulation of K in wheat significantly increased due to the application of PASP (Deng et al., 2014); PASP also increased the accumulation of K in tobacco leaves (Cao et al., 2019; Li et al., 2024; Zhang et al., 2024). Additionally, PASP improves nutrient uptake, physiological metabolism, and crop quality (Wang et al., 2018; Geng et al., 2024). Studies on corn and tobacco have found that nitrogen reduction combined with PASP application reduced nitrogen fertilizer loss and improve the nitrogen utilization efficiency (Cao et al., 2019; Tang et al., 2019a). Potassium reduction combined with PASP application also promoted the absorption and utilization of K by tobacco and increase the yield (Li et al., 2024). However, it is still unknown whether PASP can compensate for the adverse effects of K reduction on tobacco yield, quality, and post-harvest processing characteristics, and thus partially replace K fertilizer in tobacco agriculture production? It also has not been reported on how PASP affects the metabolites and microbial composition of tobacco rhizosphere soil.

Therefore, this field experiment was conducted to study effects of K fertilizer reduction and PASP application on yield, quality, processing characteristics, rhizosphere microbial composition and metabolites of flue-cured tobacco, thereby clarifying the possibility of partially replacing K fertilizer with PASP in tobacco cultivation. The study is conductive to provide guidance for alleviating the insufficient K reserves in China and mitigating the adverse effects of fertilizer application on the environment.

## 2 Materials and methods

### 2.1 Experimental materials and conduction

The field experiment was conducted in Xiangcheng county in Xuchang city of Henan province. The basic physical and chemical properties of the soil were as follows: pH 7.1; organic matter 33.0 g/kg; alkali-hydro nitrogen (N) 128.3 mg/kg; available phosphorus (P) 15.9 mg/kg; available potassium (K) 191.7 mg/kg. We set up 4 different treatments: NK (conventional fertilization as local tobacco cultivation); RK (10% reduction of K fertilizer application); NKP (4% PASP application of K_2_O amount in NK); RKP (4% PASP application of K_2_O amount in RK). The experiment designed with 3 plots (replications) for each treatment. Each plot area was 267 m² with 4 lines, a row and plant spacing of 130cm × 50cm, and the planting density of 16400 plants/hm^2^. Before transplanting, tobacco-specific compound fertilizer (17:17:17) and K_2_SO_4_ (K_2_O≥52%) were applied as basal fertilizer, and healthy tobacco seedlings (Zhongyan 100) with 4–5 true leaves were transplanted to soils. Water-soluble KNO_3_ (K_2_O≥44.5%) was equal separately supplied as topdressing using a drip irrigation system at tobacco rosette period and vigorous growth period. PASP (pH 8.5; effective content≥47%), purchased from Hebei Wozi Environmental Protection Technology Co., Ltd, was dissolved in water with KNO_3_ and irrigated to the tobacco root zone at tobacco rosette period. The application projects of fertilizers and PASP in different treatments were described in **Table 1**.

**Table 1:** Fertilization project of different treatment.

Treatment	Compound fertilizer (17:17:17) (kg/hm^2^)	K_2_SO_4_ (kg/hm^2^)	KNO_3_ (kg/hm^2^)	PASP (kg/hm^2^)
NK	750	300	37.5 + 37.5	0
RK	750	256.7	23.5 + 23.5	0
NKP	750	300	37.5 + 37.5	29.7
RKP	750	256.7	23.5 + 23.5	26.8

### 2.2 pH and nutrient content determination in soil

Before supplying basal fertilizer and after harvesting the tobacco, soil samples were collected from each plot in an “S” shape pattern at a depth of 0–20 cm. Then the soil samples were air-dried, ground, and sieved. And the pH, the content of organic matter, alkali-hydro N, available P, available K and slow-available K was quantitative determined.

### 2.3 Agronomic traits measurement

After 40 days of applying PASP through irrigation, 16 tobacco plants were randomly selected from each plot. The plant height, leaf number, leaf length, leaf width and leaf area were analyzed. Mature upper and middle tobacco leaves were harvest and bake in the bakery. Finally, the quality of flue-cured tobacco of different treatment was recorded.

### 2.4 Analysis on the physical properties of cured tobacco

First, 50 upper and middle flue-cured leaves were randomly selected from each treatment, and the vein ratio, thickness, tensile strength, elongation rate and surface density of the leaves were strict measured according to (Deng et al., 2009) and the industry standards of cured tobacco.

### 2.5 Measurement of the element content in cured tobacco

The upper and middle cured leaves were ground and sieved to determine corresponding indices. The samples were weighed and digested with concentrated H_2_SO_4_-H_2_O_2_, then the content of total N, P and K was respectively determined by fully automatic Kjeldahl nitrogen analyzer (UDK149, VELP, Italy), UV-VIS spectrophotometry (TU-1810, PGENERAL, Beijing, China), and flame spectrophotometer (FP 640, Shanghai Precision & Scientific Instrument Co., China). The content of chlorine was determined by dry ashing-potentiometric titration with silver nitrate as previous described (Kosobutskii, 2001).

### 2.6 Determination on chemical quality indicators of cured tobacco.

According to the method of Yao et al. (2010), the upper and middle cured leaves were firstly extracted in 65°C water with ethanol, and then the collected supernatant was concentrated to measure the total soluble sugar content with a high-performance liquid chromatography (HPLC, 1260, Agilent, USA). The content of reducing sugar and nicotine in cured leaves was respectively determined using 3, 5 dinitro-salicylic acid method (Wang et al., 2015) and gas chromatography-mass spectrometry (GC-MS, 7890B/5977A, Agilent, USA) (Tang et al., 2019b).

### 2.7 Metabolite analysis of rhizosphere soil

After applying PASP for 40 days, the rhizosphere soil at 5–20 cm underground in different pots was excavated using a shovel disinfected with ethanol. Each treatment was repeated 3 times. After removing visible impurities and passing through a 2-mesh sieve, 3–5 g of soil samples were taken and placed in a sterile centrifuge tube of 50 ml. Then samples were rapidly frozen with liquid nitrogen and stored in a -80°C freezer to test the metabolites.

First, vacuum freeze-drying and freeze-grinding were performed on the soil samples. Then the powder was extracted by ultrasonic extraction from an extraction solution containing methanol and centrifuged at 4°C. Finally, the supernatant was filtered through a 0.22 μm microporous membrane and the filtrate was stored in an injection bottle for Ultra High-Performance Liquid Chromatography Tandem Mass Spectrometry analysis (UPLC-MS/MS) (Agilent 1290-G6470A, Agilent Technologies, USA). The chromatographic column was Waters ACQUITY UPLCHSS T3 C18 (2.1 mm × 100 mm, 1.8 μm), the mobile phase and organic phase was ultrapure water (with 0.04% acetic acid) and acetonitrile (with 0.04% acetic acid), respectively. Mass spectrum conditions were set with a temperature of the electric spray ion source of 550°C, a mass spectrum voltage of 5500 V, and a curtain gas of 207 kPa. After obtaining metabolic spectrum data of different samples, peak area integration was performed on the mass spectrometry peaks of all substances, and the same metabolite mass spectrometry and retrograde integration were corrected for different samples. Finally, all chromatographic peak area integration data were exported and saved for quantitative analysis based on the metabolic database. Differential metabolites between different treatments were screened using the standard with |log2(Fold change) | ≥ 1.5.

### 2.8 16S rRNA sequencing and analysis

The 16S rRNA sequencing was conducted by Nanjing Sino Biotechnology Co., Ltd. (Nanjing, China). First, 0.5g of rhizosphere soil was taken and high-throughput sequencing was conducted using NovaSeq 6000 (Illumina Novaseq6000, Illumina company, San Diego, CA, USA). Then the raw sequencing data of NovaSeq 6000 were concatenated, filtered, clustered. In the meantime, the species annotation was analyzed using the Mothur method and the SSUrRNA database of SILVA138 for (with a threshold of 0.8-1), taxonomic information was obtained and the community composition of each sample was statistically analyzed at various taxonomic levels, including kingdom, phylum, class, order, family, genus, and species. Finally, non-metric multidimensional scaling (NMDS), principal component analysis (PCA), and principal coordinates analysis (PCoA) were employed to assess the effects of the experimental treatments on the composition of rhizosphere microorganism communities and ensure quality control throughout the operational process.

### 2.9 Statistical analysis on data

Data processing, statistical analysis and figure presentation were conducted by Microsoft Excel 2016, SPSS 26.0 and GraphPad Prism 10.1.2. Significant differences among the 4 treatments were determined by LSD test. Principal component analysis, heat-map analysis and multi omics correlation corplot plot were performed using the Metware Cloud, a free online platform at https://cloud.metware.cn. Different lower-case letters (a, b, c …) indicated the significant differences between the 4 treatments at the p *<* 0.05 level.

## 3 Results analysis

### 3.1 Effects of different treatments on agronomic traits of tobacco

**Figure 1:**
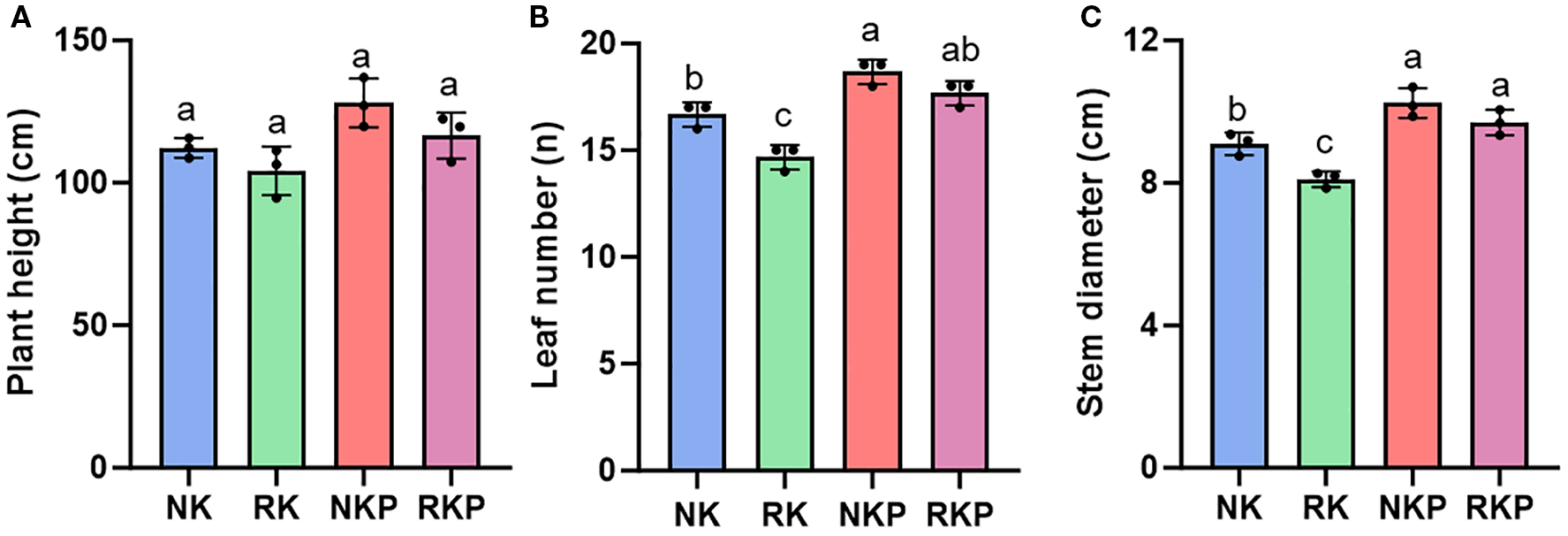
Plant parameters for different treatments. **(A)** plant height; **(B)** leaf number; **(C)** stem diameter. Different lowercase letters indicate significant differences between 4 treatments at p<0.05 level, the same as below.

As shown in **Figure 1**, compared with conventional fertilization (NK), a 10% reduction in K fertilizer (RK) had no significant effect on tobacco plant height, while reduced leaf number and stem diameter by 12.0% and 10.9%, respectively. In addition, PASP also did not affect plant height, while remarkably improved leaf number and stem diameter of tobacco no matter with normal K or reduced K application. What should be noted was that PASP application under K reduction (RKP) increased leaf number and stem diameter comparing to NK, indicating that PASP effectively compensated for the adverse effects of K fertilizer reduction on tobacco leaf number and stem thickness.

### 3.2 Effects of different treatments on the growth of leaves of tobacco

As shown in **Table 2**, compared to NK, RK had adverse effects on growth of leaves in different tobacco parts, and resulted in a deceasing of leaf length, leaf width and leaf area. In the meantime, PASP application under normal K and reduced K condition promoted growth of upper leaves and middle leaves, while had no obvious effect on leaf length, leaf width and leaf area of lower leaves, which may be attributed to that the lower leaves had completed partial growth and development when applying PAPS. What is more, compared to NK, NKP increased leaf area of upper leaves and middle leaves by 15.7% and 10.5%, while compared to RK, RKP increased leaf area of upper leaves and middle leaves by 22.2% and 11.0%. The results indicated that reducing K by 10% of conventional fertilization was not conducive to leaf growth, while the application of PSAP promoted upper and middle leaf growth at both fertilization levels, especially at 10% K reduction level.

**Table 2:** The growth of leaves in different tobacco parts.

Parts of tobacco leaves	Leaf character	Treatment			
		NK	RK	NKP	RKP
Upper leaves	Leaf length (cm)	81.2b	77.5c	85.6a	84.6a
	Leaf width (cm)	23.1ab	21.2b	25.3a	26.5a
	Leaf area (cm^2^)	1877.8c	1670.9d	2172.8a	2041.9b
Middle leaves	Leaf length (cm)	84.9bc	82.9c	86.7a	85.0b
	Leaf width (cm)	25.2ab	23.37b	28.4a	26.9ab
	Leaf area (cm^2^)	2146.5b	2000.7c	2371.3a	2219.5ab
Lower leaves	Leaf length (cm)	87.8a	84.8b	87.7a	85.1b
	Leaf width (cm)	27.3a	25.2b	28.0a	26.8b
	Leaf area (cm^2^)	2322.3b	2210.5c	2383.5a	2259.7c

Different lowercase letters indicate significant differences between 4 treatments at p<0.05 level, the same as below.

### 3.3 Effects of different treatments on the yield of flue-cured tobacco

**Figure 2:**
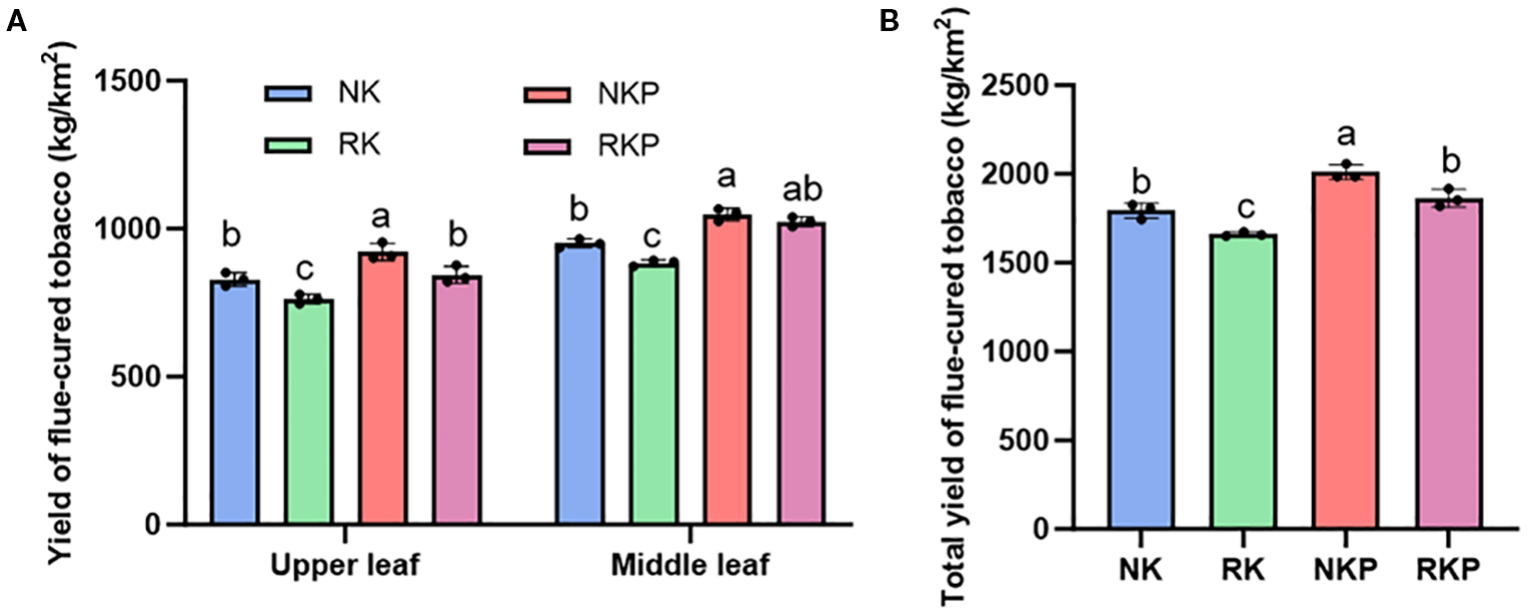
The yield of leaves in different parts **(A)** and total yield of flue-cured tobacco **(B)**.

The yield of upper and middle flue-cured leaves both was reduced by reducing of K fertilizer and the total yield decreased 133.2kg/hm^2^ (**Figure 2**). The application of PASP at NK and RK level both played an important role in increasing production of flue-cured tobacco and PASP effectively compensated for the decrease in yield of various parts of tobacco and total yield caused by K reduction. The results suggested that PASP can partially replace K fertilizer in tobacco, to a certain extent.

### 3.4 Effects of different treatments on the physical properties of cured tobacco leaves

The results of physical properties of cured tobacco leaves suggested that reducing K fertilizer by 10% improved the stem rate, while decreased the thickness, tensile strength, elongation rate, and leaf surface density of upper and middle leaves. The application of PASP was conductive to optimize physical properties, which was closely related to the elasticity and utilization rate of cured tobacco. However, compared to NKP, RKP only significantly reduced the thickness, elongation rate and leaf surface density of middle leaves, while had no effect on the physical properties of upper leaves (**Table 3**). The results indicated that applying PASP greatly alleviated the adverse effects of K reduction on the processing characteristics of flue-cured tobacco.

**Table 3:** The physical properties of cured tobacco leaves.

Parts of tobacco leaf	Treatment	Stem rate (%)	Thickness (μm)	Tensile strength (N)	Elongation rate (%)	Leaf surface density (g/m^2^)
Upper leaf	NK	23.12b	132.25b	2.37b	19.80b	104.37b
	RK	25.36a	130.40c	2.10c	17.35c	101.38c
	NKP	21.04c	136.34a	3.06a	22.96a	109.66a
	RKP	22.77bc	134.29ab	2.84a	21.84a	106.70ab
Middle leaf	NK	27.45b	126.31b	2.48b	17.94c	91.77c
	RK	29.36a	123.68c	2.26c	15.36d	86.39d
	NKP	24.85c	129.69a	2.95a	22.18a	111.54a
	RKP	25.94c	127.24b	2.74ab	20.85b	106.09b

### 3.5 Effects of different treatments on main element content in cured tobacco leaves

**Figure 3:**
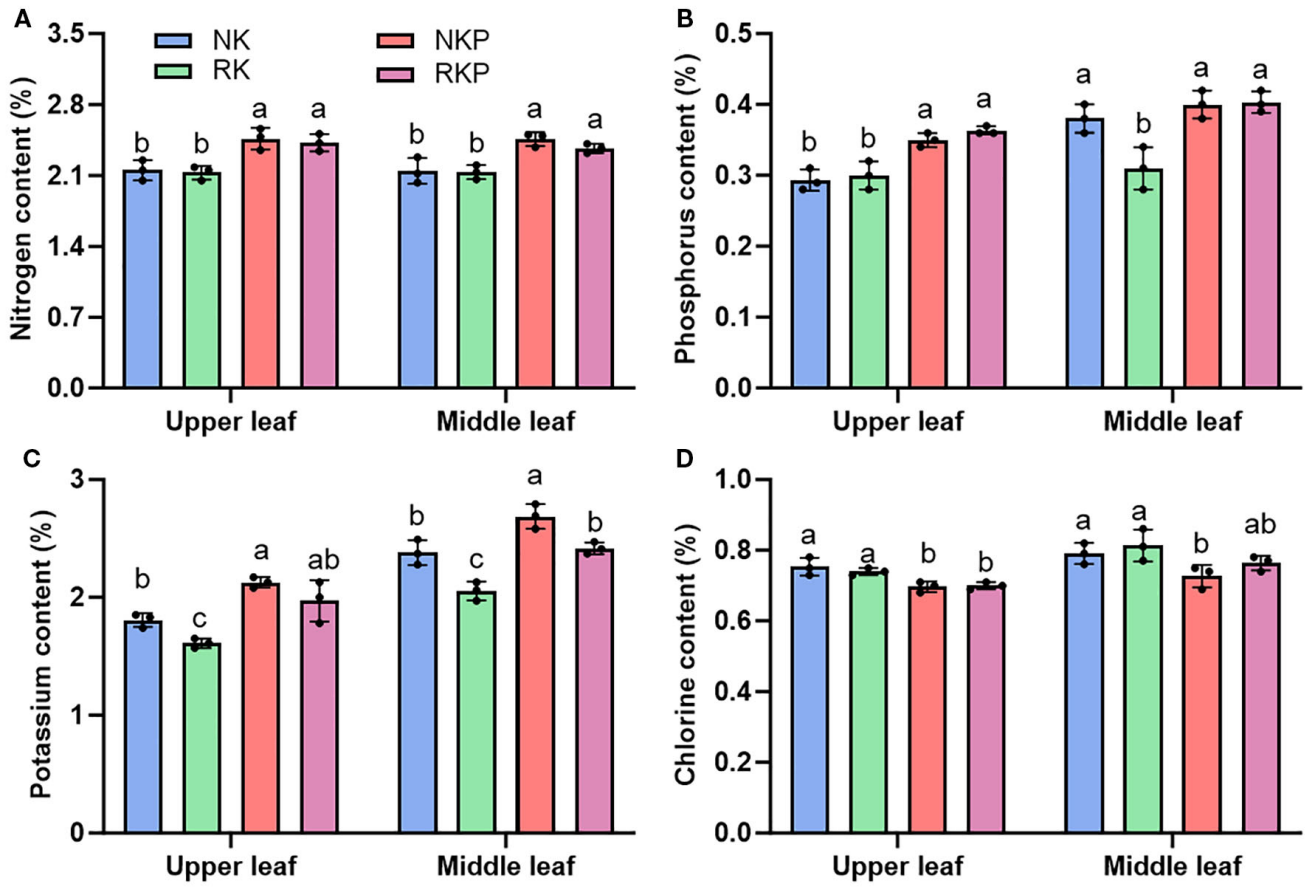
Nutrient content in upper and middle leaves for four treatments: NK, RK, NKP, and RKP. **(A)** nitrogen content; **(B)** phosphorus content; **(C)** potassium content; **(D)** chlorine content.

Reduce K fertilizer application by 10% under conventional fertilization had no effect on the content of N and Cl in upper and middle leaves, while reduced the K content in flued-cured leaves and the content of P in middle leaves (**Figure 3**). The application of PASP under different K level both increased the content of N and K and inhibited Cl accumulated in cured leaves of different parts. What is more, compared to NKP, RKP did not affect K content in upper leaves, while decreased the K content in middle leaves by 9.9%, which was lower than the decrease of NK and RK (13.7%) (**Figure 3C**). The results indicated that PASP improved the quality of cured tobacco by increasing K content and decreasing Cl content, especially under lower K fertilizer.

### 3.6 Effects of different treatments on the quality of cured tobacco leaves

As shown in **Table 4**, compared to conventional fertilization, K fertilizer reduction significantly decreased the content total sugar of flue-cured tobacco in different parts, while increased the nicotine content in middle leaves from 2.15 to 2.27. PASP application was conductive to regulate the total sugar/nicotine ratio to the suitable rage (8-10) by affecting the total sugar and nicotine. However, K application level had no effect on reducing sugar content, and PASP application had different effects on reducing sugar in tobacco of different parts, with a decreasing in upper leaves and an increasing in middle leaves. What is more, although RK decreased the nicotine and increased the total sugar/nicotine ratio comparing to NK, RPK and NPK had no obvious difference on nicotine content and the total sugar/nicotine ratio, suggesting that PASP compensated the adverse effect on the nicotine and total sugar/nicotine induced by K reduction.

**Table 4:** The quality indices of flue-cured tobacco leaves.

Parts of tobacco leaf	Treatment	Total sugar content (%)	Reducing sugar content (%)	Nicotine content (%)	Total sugar/nicotine
Upper leaf	NK	24.42b	23.51a	2.23b	19.93a
	RK	23.29c	24.16a	2.26b	10.33b
	NKP	27.81a	21.60b	2.76a	9.85c
	RKP	26.14b	22.24b	2.68a	9.73c
Middle leaf	NK	23.12b	21.35b	2.15b	10.73a
	RK	22.02c	21.40b	2.27c	10.70a
	NKP	25.75a	22.95a	2.59a	9.91b
	RKP	24.77ab	21.74b	2.49a	9.96b

### 3.7 Effects of different treatments on the nutrient content in soil after tobacco harvest

Reduced supply of K fertilizer inhibited the degradation of organic matter and decreased the content of alkali-hydro N, available P and K, while did not affect slow-available K content in soil. The application of PASP reduced the content of organic matter and slow-available K in soil (**Table 5**). Although PASP promoted the absorption on N and K of tobacco, the higher content of alkali-hydro N, available K and lower content of slow-available K indicated that PASP promoted the degradation of organic matter to alkali-hydro N and the transformation of slow-available K to available K.

**Table 5:** The nutrient content in soil.

Treatment	Organic matter (g/kg)	Alkali-hydro N (mg/kg)	Available P (mg/kg)	Available K (mg/kg)	Slow-available K (mg/kg)
NK	13.25b	112.12b	15.81a	119.25b	486.32a
RK	15.36a	97.46c	13.29b	108.36c	481.65a
NKP	11.23c	130.03a	14.80a	127.09a	451.56b
RKP	12.55b	116.79b	15.14a	122.89ab	462.39b

### 3.8 Effects of different treatments on the metabolite components in rhizosphere

**Figure 4:**
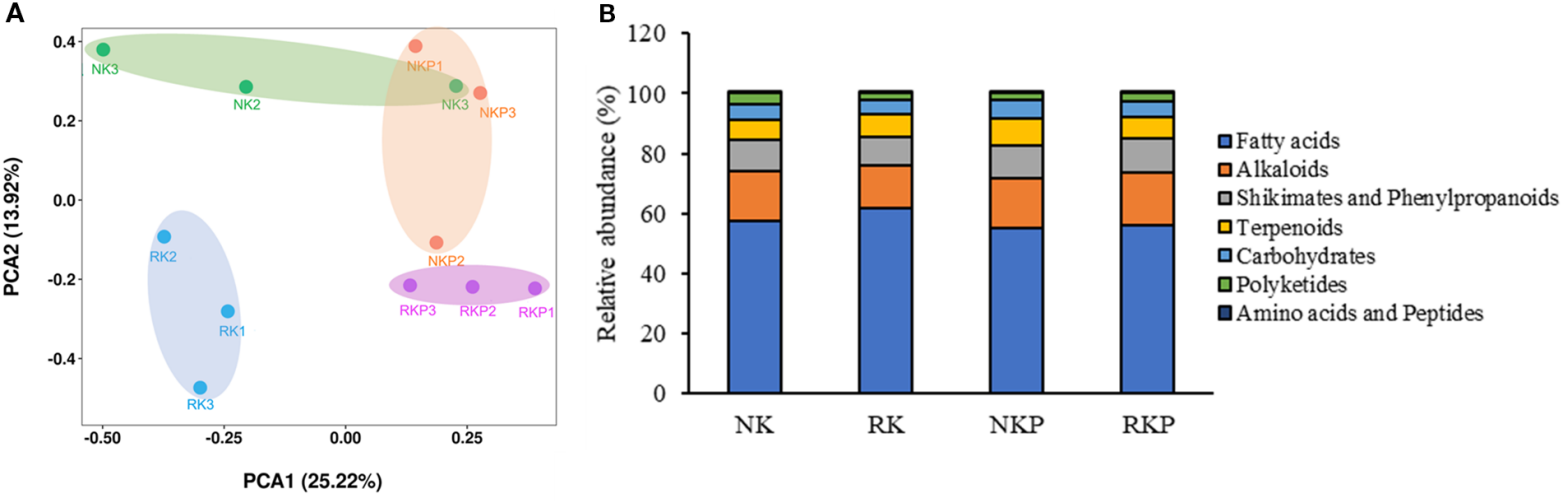
Metabolomics analysis of tobacco rhizosphere soil under different treatments. **(A)** Principal component analysis of rhizosphere metabolites; **(B)** Distribution of different metabolite components in rhizosphere metabolism.

Through principal component analysis (PCA) and of metabolites in rhizosphere soil of four different treatments, we found that reduction of K fertilizer (RK) and traditional fertilization (NK) had significant difference in PCA2 (13.92%), and PASP application under K fertilizer reduction (RKP) obvious affected the metabolites in rhizosphere in PCA1 (25.22%), while PASP application under traditional fertilization (NKP) did not affect the rhizosphere metabolites (**Figure 4A**). The metabolites were furtherly divided into 7 groups: fatty acids, alkaloids, shikimates and phenylpropanoids, terpenoids, carbohydrates, polyketides, amino acids and peptides (**Figure 4B**). The results showed that compared to NK, RK increased the relative abundance of fatty acids and terpenoids, while decreased the relative abundance of alkaloids, shikimates and phenylpropanoids, and polyketides. Compared to NK, NKP increased the relative abundance of polyketides and terpenoids; compared to RK, RKP increased the relative abundance of alkaloids, shikimates and phenylpropanoids, polyketides, and decreased the relative abundance of fatty acids and terpenoids; compared to NKP, RKP increased the relative abundance of alkaloids, and decreased the relative abundance of terpenoids and carbohydrates.

### 3.9 Analysis on the differential metabolites in rhizosphere

**Figure 5:**
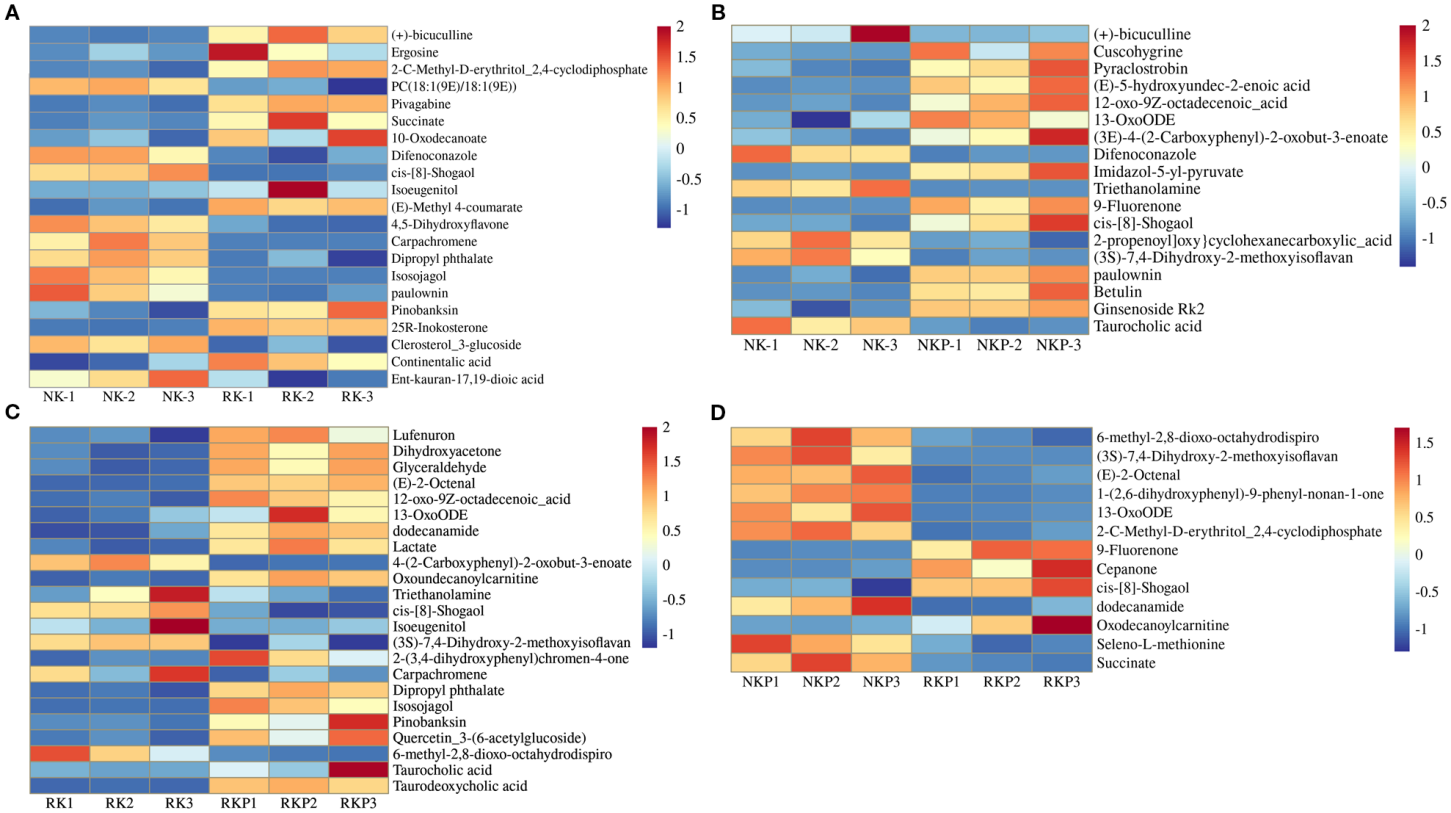
Thermographic analysis of differential metabolites in the rhizosphere between different treatments. **(A)** Differential metabolites of K fertilizer reduction treatment and normal K fertilizer treatment. **(B)** Differential metabolites of normal K fertilizer + PASP application treatment and normal K fertilizer treatment. **(C)** Differential metabolites of K fertilizer reduction + PASP application treatment and K fertilizer reduction treatment. **(D)** Differential metabolites of K fertilizer reduction + PASP application treatment and normal K fertilizer + PASP application treatment.

**Figure 6:**
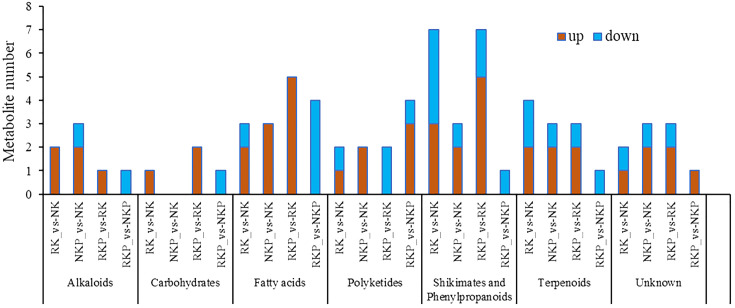
Changes in different metabolite components of rhizosphere between different treatments.

To study the effects of K reduction and PASP application on the metabolic process of tobacco roots, we further analyzed the changes in the relative content of differential metabolites of the 7 components (**Figures 5**, **6**). Compared to NK, RK increased the content of 11 metabolites, including 2 alkaloids (bicuculline, ergosine), 1 carbohydrate (2-C-Methyl-D-erythritol 2,4-cyclodiphosphate), 2 fatty acids (pivagabine, succinate), 2 shikimates and phenylpropanoids (Methyl 4-coumarate, pinobanksin), 2 terpenoids (25R-Inokosterone, continentalic acid), 1 polyketide (isoeugenitol), and 1 unknown metabolite (10-Oxodecanoate), while decreased 10 metabolites, including 1 fatty acid (glycerophosphocholines), 5 shikimates and phenylpropanoids (4,5-Dihydroxyflavone, carpachromene, isosojagol, paulownin, dipropyl phthalate), 2 terpenoids (clerosterol 3-glucoside, ent-kauran-17,19-dioic acid), 1 polyketide (cis-[8]-Shogaol), and 1 unknown metabolite (difenoconazole) (**Figures 5A**, **6**). Under normal K condition, PASP application increased 12 metabolites, including 1 alkaloid (cuscohygrine), 3 fatty acids (5-hydroxyundec-2-enoic acid, 12-oxo-9Z-octadecenoic acid, 13-OxoODE), 1 shikimate and phenylpropanoid (paulownin), 2 terpenoids (betulin, ginsenoside Rk2), 2 polyketides (9-Fluorenone, cis-[8]-Shogaol), and 3 unknown metabolites (pyraclostrobin, (3E)-4-(2-Carboxyphenyl)-2-oxobut-3-enoate, imidazol-5-yl-pyruvate), while decreased 1 alkaloid (bicuculline), 2 shikimates and phenylpropanoids (2-propenoyloxy cyclohexanecarboxylic acid, (3S)-7,4-Dihydroxy-2-methoxyisoflavan), 1 terpenoid (taurocholic acid), and 2 unknown metabolites (difenoconazole, triethanolamine) (**Figures 5B**, **6**). Under K reduction, PASP application affected more metabolites in rhizosphere, resulting in increases on 2 alkaloids (lufenuron, lactate), 2 carbohydrates (dihydroxyacetone, glyceraldehyde), 4 fatty acids (dodecanamide, 12-oxo-9Z-octadecenoic acid, 13-OxoODE, (E)-2-Octenal), 5 shikimates and phenylpropanoids (quercetin 3-(6-acetylglucoside), 2-(3,4-dihydroxyphenyl) chromen-4-one, dipropyl phthalate, isosojagol, pinobanksin), 2 terpenoids (taurocholic acid, taurodeoxycholic acid), and 1 unknown metabolite: (3E)-4-(2-Carboxyphenyl)-2-oxobut-3-enoate, while resulting in decreases on 1 shikimate and phenylpropanoid: (3S)-7,4-Dihydroxy-2-methoxyisoflavan, 1 terpenoid: 6-methyl-2,8-dioxo-octahydrodispiro, 2 polyketides (cis-[8]-Shogaol, isoeugenitol), and 2 unknown metabolites (triethanolamine, oxoundecanoylcarnitine) (**Figures 5C**, **6**). Interestingly, under normal and reduced K condition, PASP application only affected 13 metabolites (**Figure 5D**), indicating that PASP relieved the impact of K reduction on the metabolite composition of tobacco rhizosphere soil. The results suggested that PASP application under K reduction had more obvious effects on the metabolites in rhizosphere of tobacco.

### 3.10 Correlation analysis between rhizosphere differential metabolites and tobacco yield, quality, and physical characteristics

**Figure 7:**
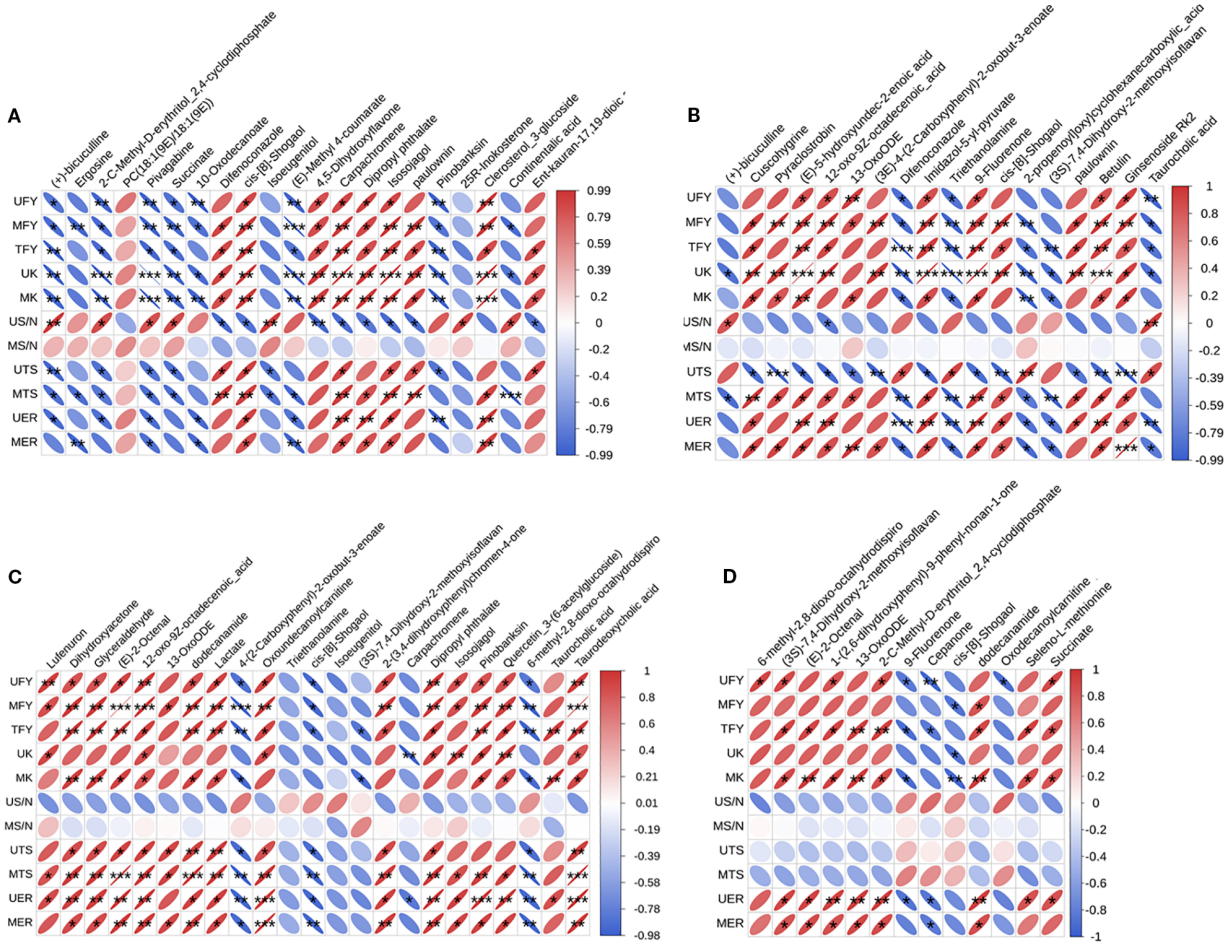
Correlation analysis of rhizosphere differential metabolites and rhizosphere soil nutrients, flue-cured tobacco yield and quality between **(A)** K fertilizer reduction treatment and normal K fertilizer treatment; **(B)** normal K fertilizer + PASP application treatment and normal K fertilizer treatment; **(C)** K fertilizer reduction + PASP application treatment and K fertilizer reduction treatment; **(D)** K fertilizer reduction + PASP application treatment and normal K fertilizer + PASP application treatment. UFY, MFY and TFY represent the yield of upper, middle, and total flue-cured tobacco; UK, MK represent K content in upper and middle cured leaf; US/N, MS/N represent sugar/nicotine ratio in upper and middle cured leaf; UTS, MTS represent total sugar content in upper and middle cured leaf; UER, MER represent elongation rate of upper and middle cured leaf.

To further investigate the potential mechanisms by which K reduction and PASP affect the yield, chemical and physical quality of flue-cured tobacco, we conducted correlation analyses focusing on rhizosphere differential metabolites and tobacco yield, total sugar/nicotine, total sugar content and elongation rate. As illustrated in **Figure 7**, the yield and K content of flue-cured tobacco exhibited significant negative correlation with bicuculline, 2-C-Methyl-D-erythritol 2,4-cyclodiphosphate, 10-Oxodecanoate, pivagabine, (E)-Methyl 4-coumarate, pinobanksin, triethanolamine, taurocholic acid, 6-methyl-2,8-dioxo-octahydrodispiro, 9-Fluorenone and oxodecanoylcarnitine, while exhibited significant active correlation with 4,5-Dihydroxyflavone, carpachromene, dipropyl phthalate, paulownin, isosojagol, clerosterol 3-glucoside, cuscohygrine, pyraclostrobin, (E)-5-hydroxyundec-2-enoic acid, 12-oxo-9Z-octadecenoic acid, imidazol-5-yl-pyruvate, botulin, ginsenoside Rk2, lufenuron, dihydroxyacetone, glyceraldehyde, dodecanamide, lactate, 2-(3,4-dihydroxyphenyl)chromen-4-one, and quercetin 3-(6-acetylglucoside). In the meantime, although total sugar content of upper and middle leaf was closely correlated to rhizosphere differential metabolites, only the ratio of total sugar and nicotine of upper leaf had significantly correction with bicuculline, pivagabine, 4,5-Dihydroxyflavone, 25R-Inokosterone, continentalic acid, and ent-kauran-17,19-dioic acid. The results also showed a relatively high correlation between 18 kinds of rhizosphere differential metabolites and elongation rate of flue-cured tobacco.

### 3.11 Diversity and composition of microorganism in rhizosphere soil responding to K reduction and PASP application

**Figure 8:**
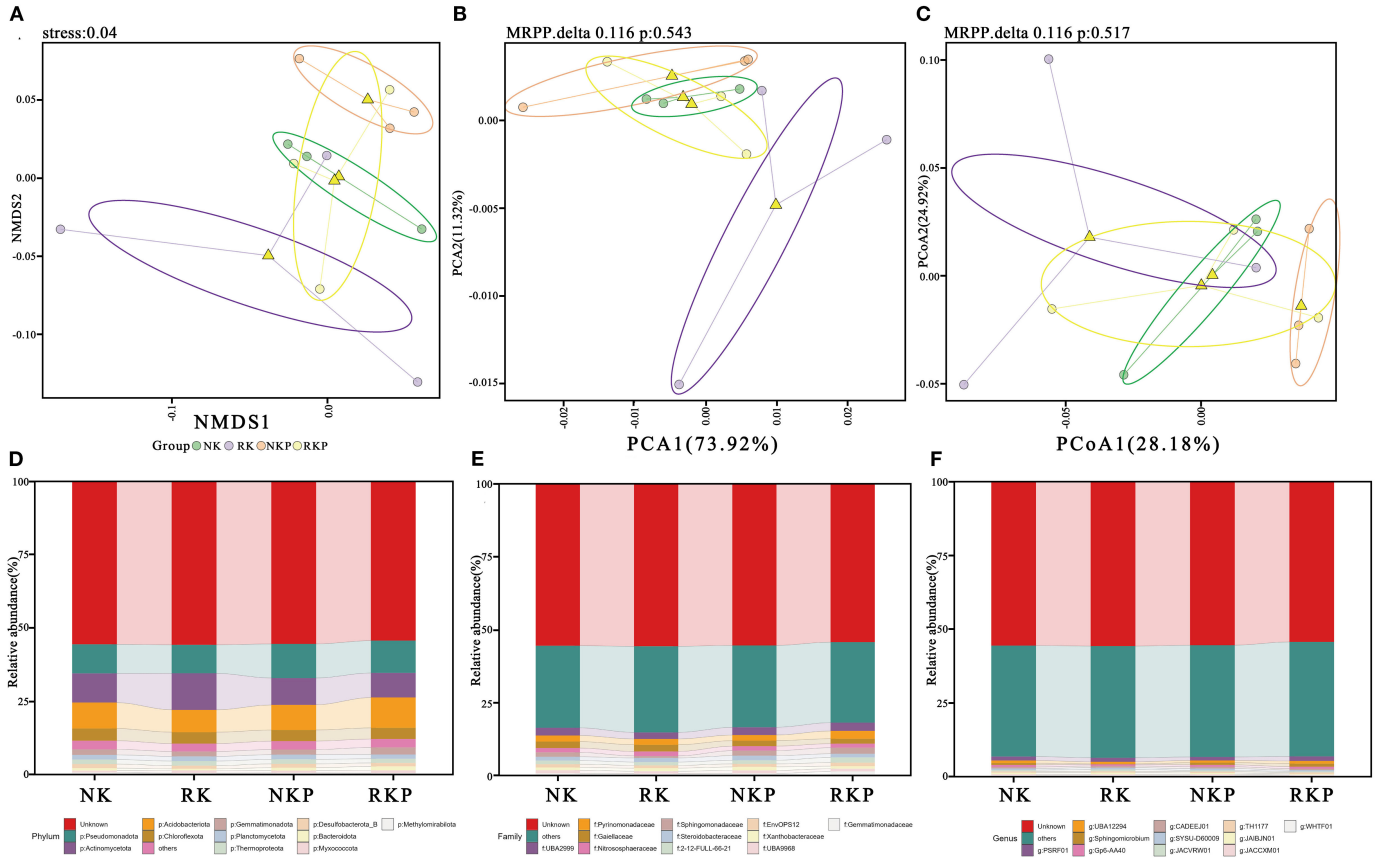
Non-metric multidimensional scaling (NMDS, **(A)**, principal component analysis (PCA, **(B)**, principal coordinates analysis (PCoA, **(C)** and relative abundance **(B)** of the rhizosphere microorganism at the phylum **(D)**, family **(E)** and genus **(F)** level.

To investigate the response of the rhizosphere microorganism community to K reduction and PASP, we constructed rhizosphere soil 16S rRNA amplification libraries under different conditions, followed by Illumina sequencing. Dimensionality reduction analysis of species diversity, including non-metric multidimensional scaling (NMDS), principal component analysis (PCA), and principal coordinates analysis (PCoA) both revealed not clear separations of samples across the four treatments (**Figures 8A–C**). The statistical analysis of the relative abundance of rhizosphere soil microorganisms at various taxonomic levels (Phylum, Family, Genus) also showed no significant difference in species diversity among the treated samples (**Figures 8D–F**). These findings suggest that the rhizosphere microorganism community had no markedly distinct responses to K reduction and PASP treatments.

## 4 Discussions

### 4.1 Potassium reduction and PASP application affected tobacco yield and processing characteristics

Potassium is important to improve quality of crops including sugarcane (Bhatt et al., 2023; Kumar et al., 2024), cotton (Zahoor et al., 2017), tobacco (Hu et al., 2021), and orange (Wu et al., 2024). It is urgent to resolve the contradiction between the preference for K in crops and the insufficient K mineral resources in China. Potassium deficiency not only inhibited leaf and root development of tobacco (Song et al., 2015; Liu et al., 2020), but also limited the quality, processing characteristics and taste of flue-cured tobacco (Zhang et al., 2016; Wen et al., 2018). Our study suggested reducing K fertilizer by 10% inhibited leaf growth, reduced the yield and utilization rate of flue-cured tobacco by decreasing thickness, tensile strength, elongation, surface density and increasing stem rate, while PASP application promoted growth of tobacco, increased the yield and post-harvest processing characteristics. It has been reported that PASP application under K reduction promoted K absorption and utilization and raised the yield of flue-cured tobacco (Li et al., 2024), and our results also indicated that PASP activated slow-release K in soil to increase available K content, thereby compensated the adverse effect on K content in upper and middle leaves induced by K reduction. our study found for the first time that PASP reduced the stem rate, increased the thickness, tensile strength, elongation rate, and leaf density of tobacco, compensating for the decrease in physical characteristics of tobacco caused by a 10% reduction in K fertilizer application, which may be attributed to PASP promoting the absorption and utilization of K (Qiao et al., 2020; Zhang et al., 2024) and photosynthesis (Cao et al., 2019; Chen et al., 2024).

### 4.2 Potassium reduction and PASP application affected chemical quality of tobacco

What is more, K reduction decreased the content of total sugar in leaves of different parts of tobacco, which may be attributed to weakened photosynthesis (Lu et al., 2019; Ladikou et al., 2025). The nicotine content in tobacco leaves was negatively correlated with K content duo to the inhibition of K on activity of nicotine synthases (arginine decarboxylase: ADC; ornithine decarboxylase: ODC) (Wang et al., 2012) and expression of *PMT* (putrescine N-methyltransferase) (Watson and Malmberg, 1996; Li et al., 2019). However, although PASP application under normal and reduced K conditions both promoted K accumulation in leaves of different parts, the nicotine content did not decrease but increase with increasing of K content. This phenomenon may be attributed to the elevated nitrogen absorption induced by PASP, which subsequently enhanced nicotine biosynthesis (Yang et al., 2024, Yang et al., 2025). Sugar nicotine ratio depends on the total sugar and nicotine content and suitable sugar nicotine ratio (8-10) is contributed to balance the taste and stimulation of tobacco (Wu et al., 2006). The higher sugar/nicotine in upper and middle tobacco leaves of different K conditions was reduced to an appropriate range due to PASP application, indicated that the increasing effect of PASP on nicotine content was greater than on total sugar content. However, our previous study had found although PASP reduced sugar/nicotine, it had different effect on the sugar and nicotine content in tobacco leaves of varied parts (Zhang et al., 2024). Therefore, it is necessary to further elucidate the intrinsic mechanism by which PASP raises the sugar nicotine ratio of different parts of tobacco.

### 4.3 The effects of K reduction and PASP application on soil microbial community and metabolite composition

The rhizosphere soil is the most critical area for plant growth and a hot topic in nutrient transformation and soil microbial community research. It has been reported that 10% of carbon fixed by net photosynthesis can be released as root exudates into the rhizosphere, thereby influencing nutrient uptake, soil microbial community diversity, composition, and network complexity (Lareen et al., 2016; Jiang et al., 2024). 10% reduction of K fertilizer and PASP significant changed the concentration and composition of metabolites in rhizosphere. Our findings highlighted the significant impact of rhizosphere metabolites on tobacco yield, K uptake and quality. The soil microorganism’s composition, diversity, and abundance are closely related to soil functions, plant growth and productivity by producing the signaling compounds (Ganugi et al., 2022; Zheng et al., 2018). Studies on tobacco (Jiao et al., 2024) and sugarcane (Zhang et al., 2025) showed that K affected the structure of soil microbial community and the soil microbial diversity, and PASP also can reshape soil microbiome in rice soil (Liu et al., 2023). However, our study suggested that 10% reduction of K and 4% application of PASP had no significant effect on microorganism composition in rhizosphere soil of tobacco in field, which may be due to insufficient K reduction and PASP application or shorter treatment time. Further experiment should set higher K reductions and PASP applications and determine soil microbial community in different growth stages.

## 5 Conclusions

Our study revealed that K reduction inhibited tobacco growth, decreased the yield, positively affected physical and chemical quality, and changed metabolite composition in rhizosphere soil, while PASP application played a significant role in promoting leaf growth, K absorption, coordinating sugar/nicotine and improving processing characteristics of flue-cured tobacco. PASP application under K reduction was beneficial for compensating for the adverse effects of K reduction on tobacco growth, K absorption, yield, and quality, thereby replacing K fertilizer to a certain extent to reduce the application of K fertilizer in agricultural production. The findings indicated the importance of metabolites composition in driving tobacco yield and quality, thereby providing valuable insights for future production practices and suggest practical management strategies that apply specific metabolites to optimize tobacco performance. This study also provided a theoretical basis and scientific guidance for saving K mineral resources and alleviating environmental pressure in China.

## Data Availability

The original contributions presented in the study are included in the article/**Supplementary Material**. Further inquiries can be directed to the corresponding author/s.
