# Supplementary material for: Effects of potassium fertilizer reduction combined with polyaspartic acid application on yield, quality, processing characteristics, rhizosphere microbial composition and metabolites of flue-cured tobacco
Source: Front Plant Sci. 2025 Sep 19;16:1672845. doi: 10.3389/fpls.2025.1672845 (PMC12491321; doi:10.3389/fpls.2025.1672845)
Supplement: Supplementary file 1 [file Table1.docx]

Table S1 The differential metabolites in rhizosphere soil of tobacco between varied treatments

|  | ID | Regulated | Class | Super class |
| --- | --- | --- | --- | --- |
| *RK_ VS _NK* | (+)-Bicuculline | Up | Tyrosine alkaloids | Alkaloids |
|  | Ergosine | Up | Tryptophan alkaloids |  |
|  | 2-C-Methyl-D-erythritol 2,4-cyclodiphosphate | Up | Saccharides | Carbohydrates |
|  | Glycerophosphocholines | Down | Glycerophospholipids | Fatty acids |
|  | Pivagabine | Up | Fatty acids and conjugates |  |
|  | Succinate | Up | Fatty acids and conjugates |  |
|  | cis-[8]-Shogaol | Down | Aromatic polyketides | Polyketides |
|  | Isoeugenitol | Up | Chromanes |  |
|  | (E)-Methyl 4-coumarate | Up | Phenylpropanoids | Shikimates and Phenylpropanoids |
|  | 4,5-Dihydroxyflavone | Down | Flavonoids |  |
|  | Carpachromene | Down | Flavonoids |  |
|  | Dipropyl phthalate | Down | Phenolic acids |  |
|  | Isosojagol | Down | Isoflavonoids |  |
|  | Paulownin | Up | Lignans |  |
|  | Pinobanksin | Up | Flavonoids |  |
|  | 25R-Inokosterone | Up | Steroids | Terpenoids |
|  | Clerosterol 3-glucoside | Down | Steroids |  |
|  | Continentalic acid | Up | Diterpenoids |  |
|  | Ent-kauran-17, 19-dioic acid | Down | Diterpenoids |  |
|  | 10-Oxodecanoate | Up | Unknown | Unknown |
|  | Difenoconazole | Down | Unknown |  |
| *NK_ VS* *_NKP* | (+)-Bicuculline | Down | Tyrosine alkaloids | Alkaloids |
|  | Cuscohygrine | Up | Ornithine alkaloids |  |
|  | Pyraclostrobin | Up | Unknown |  |
|  | (E)-5-hydroxyundec-2-enoic acid | Up | Fatty acids and conjugates | Fatty acids |
|  | 12-oxo-9Z-octadecenoic acid | Up | Fatty acids and conjugates |  |
|  | 13-OxoODE | Up | Fatty acids and conjugates |  |
|  | 9-Fluorenone | Up | Polycyclic aromatic polyketides | Polyketides |
|  | cis-[8]-Shogaol | Up | Aromatic polyketides |  |
|  | 2-propenoyl cyclohexanecarboxylic acid | Down | Phenylpropanoids | Shikimates and phenylpropanoids |
|  | (3S)-7,4-Dihydroxy-2-methoxyisoflavan | Down | Isoflavonoids |  |
|  | Paulownin | Up | Lignans |  |
|  | Betulin | Up | Triterpenoids | Terpenoids |
|  | Ginsenoside | Up | Triterpenoids |  |
|  | Taurocholic acid | Down | Steroids |  |
|  | (3E)-4-(2-Carboxyphenyl)-2-oxobut-3-enoate | Down | Unknown | Unknown |
|  | Difenoconazole | Up | Unknown |  |
|  | Imidazol-5-yl-pyruvate | Down | Unknown |  |
|  | Triethanolamine | Up | Unknown |  |
| *RKP_ VS* *_RK* | Lufenuron | Up | Unknown | Alkaloids |
|  | Dihydroxyacetone | Up | Saccharides | Carbohydrates |
|  | Glyceraldehyde | Up | Saccharides |  |
|  | (E)-2-Octenal | Up | Fatty acyls | Fatty acids |
|  | 12-oxo-9Z-octadecenoic acid | Up | Fatty acids and conjugates |  |
|  | 13-OxoODE | Up | Fatty acids and conjugates |  |
|  | Dodecanamide | Up | Fatty amides |  |
|  | Lactate | Up | Fatty acids and conjugates |  |
|  | Triethanolamine | Down | Aromatic polyketides | Polyketide |
|  | cis-[8]-Shogaol | Down | Chromanes |  |
|  | Isoeugenitol | Down | Isoflavonoids | Shikimates and phenylpropanoids |
|  | (3S)-7,4-Dihydroxy-2-methoxyisoflavan | Up | Flavonoids |  |
|  | 2-(3,4-dihydroxyphenyl) chromen-4-one | Down | Flavonoids |  |
|  | Carpachromene | Up | Phenolic acids |  |
|  | Dipropyl phthalate | Up | Isoflavonoids |  |
|  | Isosojagol | Up | Flavonoids |  |
|  | Pinobanksin | Up | Flavonoids |  |
|  | Quercetin 3-(6-acetylglucoside) | Up | Diterpenoids | Terpenoids |
|  | 6-methyl-2,8-dioxo-octahydrodispiro | Down | Steroids |  |
|  | Taurocholic acid | Up | Steroids |  |
|  | Taurodeoxycholic acid | Up | Unknown | Unknown |
|  | (3E)-4-(2-Carboxyphenyl)-2-oxobut-3-enoate | Up | Unknown |  |
|  | Oxoundecanoylcarnitine | Down | Unknown |  |
| *RKP_ VS _NKP* | 6-methyl-2,8-dioxo-octahydrodispiro | Down | Small peptides | Amino acids and peptides |
|  | (3S)-7,4-Dihydroxy-2-methoxyisoflavan | Down | Saccharides | Carbohydrates |
|  | (E)-2-Octenal | Down | Fatty acyls | Fatty acids |
|  | 1-(2,6-dihydroxyphenyl)-9-phenyl-nonan-1-one | Down | Fatty acids and conjugates |  |
|  | 13-OxoODE | Down | Fatty amides |  |
|  | 2-C-Methyl-D-erythritol-2,4-cyclodiphosphate | Down | Fatty acids and conjugates |  |
|  | Cepanone | Up | Polycyclic aromatic polyketides | Polyketides |
|  | cis-[8]-Shogaol | Up | Aromatic polyketides |  |
|  | Dodecanamide | Down | Polycyclic aromatic polyketides |  |
|  | Oxodecanoylcarnitine | Up | Cyclic polyketides |  |
|  | Seleno-L-methionine | Down | Aromatic polyketides | Shikimates and phenylpropanoids |
|  | Succinate | Down | Isoflavonoids | Terpenoids |
|  | 9-Fluorenone | Up | Unknown | Unknown |
